# 3-Bromo-7-meth­oxy-2-phenyl­imidazo[2,1-*b*][1,3]benzothia­zole

**DOI:** 10.1107/S1600536813006582

**Published:** 2013-03-13

**Authors:** Alexander S. Bunev, Elena V. Sukhonosova, Dinara R. Syrazhetdinova, Vladimir E. Statsyuk, Gennady I. Ostapenko, Victor N. Khrustalev

**Affiliations:** a Department of Chemistry and Chemical Technology, Togliatti State University, 14 Belorusskaya Street, Togliatti 445667, Russian Federation; b Department of Bio­organic and Medicinal Chemistry, Samara State University, 1 Academika Pavlova Street, Samara 443011, Russian Federation; c X-ray Structural Centre, A. N. Nesmeyanov Institute of Organoelement Compounds, Russian Academy of Sciences, 28 Vavilov Street, B-334, Moscow 119991, Russian Federation

## Abstract

In the title mol­ecule, C_16_H_11_BrN_2_OS, the central imidazo[2,1-*b*][1,3]benzothia­zole tricycle is essentially planar (r.m.s. deviation = 0.021 Å). The terminal phenyl ring is twisted at 36.18 (5)° from the mean plane of the tricycle. In the crystal, pairs of eak C—H⋯O hydrogen bonds link mol­ecules into centrosymmetric dimers, which are further packed into stacks along the *a* axis.

## Related literature

For applications of imidazo[2,1-*b*][1,3]benzothia­zoles, see: Mase *et al.* (1988 ▶); Ager *et al.* (1988 ▶); Barchéchath *et al.* (2005 ▶); Kumbhare *et al.* (2011 ▶); Yousefi *et al.* (2011 ▶); Chandak *et al.* (2013 ▶). For the crystal structures of related compounds, see: Landreau *et al.* (2002 ▶); Adib *et al.* (2008 ▶); Fun, Asik *et al.* (2011 ▶); Fun, Hemamalini *et al.* (2011 ▶).
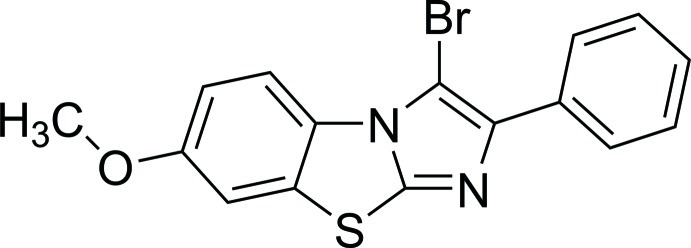


## Experimental

### 

#### Crystal data

C_16_H_11_BrN_2_OS
*M* *_r_* = 359.24
Monoclinic, 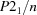

*a* = 3.8346 (4) Å
*b* = 9.4848 (11) Å
*c* = 37.236 (4) Å
β = 91.810 (2)°
*V* = 1353.6 (3) Å^3^
*Z* = 4
Mo *K*α radiation
μ = 3.19 mm^−1^
*T* = 100 K
0.35 × 0.15 × 0.15 mm

#### Data collection

Bruker APEXII CCD diffractometer
Absorption correction: multi-scan (*SADABS*; Sheldrick, 2003 ▶) *T*_min_ = 0.402, *T*_max_ = 0.646
17224 measured reflections
3924 independent reflections
3578 reflections with *I* > 2σ(*I*)
*R*_int_ = 0.041

#### Refinement

*R*[*F* ^2^ > 2σ(*F* ^2^)] = 0.032
*wR*(*F* ^2^) = 0.070
*S* = 1.00
3924 reflections
191 parameters
H-atom parameters constrained
Δρ_max_ = 0.59 e Å^−3^
Δρ_min_ = −0.95 e Å^−3^

### 

Data collection: *APEX2* (Bruker, 2005 ▶); cell refinement: *SAINT* (Bruker, 2001 ▶); data reduction: *SAINT*; program(s) used to solve structure: *SHELXTL* (Sheldrick, 2008 ▶); program(s) used to refine structure: *SHELXTL*; molecular graphics: *SHELXTL*; software used to prepare material for publication: *SHELXTL*.

## Supplementary Material

Crystal structure: contains datablock(s) global, I. DOI: 10.1107/S1600536813006582/cv5391sup1.cif

Structure factors: contains datablock(s) I. DOI: 10.1107/S1600536813006582/cv5391Isup2.hkl

Supplementary material file. DOI: 10.1107/S1600536813006582/cv5391Isup3.cml

Additional supplementary materials:  crystallographic information; 3D view; checkCIF report

## Figures and Tables

**Table 1:** Hydrogen-bond geometry (Å, °)

*D*—H⋯*A*	*D*—H	H⋯*A*	*D*⋯*A*	*D*—H⋯*A*
C8—H8⋯O1^i^	0.95	2.56	3.465 (3)	158

Symmetry code: (i) 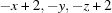.

**Fig. 1.**
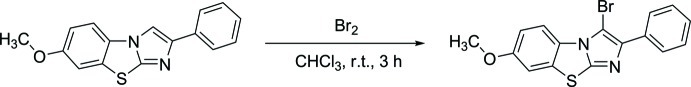
The reaction of imidazo[2,1-b][1,3]benzothiazoles with bromine.

**Fig. 2.**
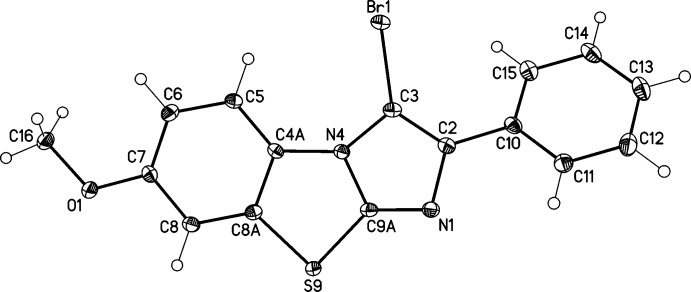
Molecular structure of I. Displacement ellipsoids are shown at the 50% probability level. H atoms are presented as small spheres of arbitrary radius.

**Fig. 3.**
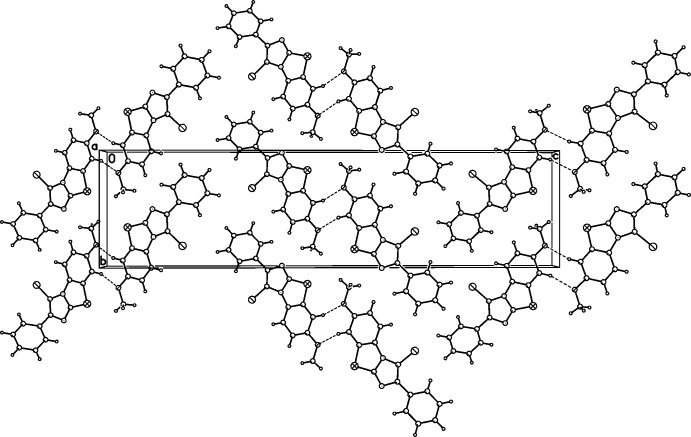
A portion of the crystal structure showing the packing of the H-bonded centrosymmetrical dimers of I. The weak intermolecular C—H···O hydrogen bonds are drawn by dashed lines.

## supplementary crystallographic information

### Comment

Imidazo[2,1–*b*][1,3]benzothiazoles are of great interest due to their biological properties. These compounds and their derivatives demonstrate the immunosuppressive (Mase *et al.*, 1988), antiallergic (Ager *et al.*, 1988) and anti-cancer (Kumbhare *et al.*, 2011) activities as well as the inhibition activity of apoptosis in testiculargerm cells (Chandak *et al.*, 2013) and lymphocytes (Barchéchath *et al.*, 2005). Moreover, the substituted imidazo[2,1–*b*][1,3]benzothiazoles have emerged as significant components in various diversified therapeutic applications. In particular, they are potential agents for High-Contrast PET Imaging (Yousefi *et al.*, 2011). In this work, a new halogensubstituted imidazo[2,1-*b*][1,3]benzothiazole, C_16_H_11_N_2_OSBr, (I) was prepared by the reaction of imidazo[2,1–*b*][1,3]benzothiazole with bromine at room temperature (Figure 1), and its structure was unambiguously established by the X-ray diffraction study (Figure 2).

The bond lengths and angles within the molecule of I are in a good agreement with those found in the related compounds (Landreau *et al.*, 2002; Adib *et al.*, 2008; Fun, Asik *et al.*, 2011; Fun, Hemamalini *et al.*, 2011). The central imidazo[2,1–*b*][1,3]benzothiazole fragment is essentially planar (r.m.s. deviation is 0.021 Å). The methoxy group is practically coplanar to this fragment (the corresponding C6—C7—O1—C16 dihedral angle is 1.8 (3)°), and the terminal phenyl ring is twisted from it at 36.18 (5)°.

In the crystal, molecules form the centrosymmetrical dimers by the weak intermolecular C—H···O hydrogen bonds (Table 1, Figure 3). The dimers are further packed into stacks along the *a* axis.

### Experimental

A solution of bromine (0.51 ml, 1.6 g, 10 mmol) in dry CHCl_3_ (10 ml) was added to a solution of 7-methoxy-2-phenylimidazo[2,1-*b*][1,3]benzothiazole (2.80 g, 10 mmol) in dry CHCl_3_ (50 ml). The reaction mixture was stirred at room temperature for 3 h. The solvent was evaporated from the reaction mixture on rotavapor. The crude product was diluted with 5% solution of Na_2_CO_3_ in water (50 ml). The precipitate was filtered and crystallized from dimethylformamide. Yield is 89%. The single crystals of I were obtained by slow crystallization from dimethylformamide. *M*.p. = 447–448 K. IR (KBr), ν/cm^-1^: 2961, 1607, 1580, 1493, 1222, 684,633. ^1^H NMR (500 MHz, DMSO-*d*_6_, 303 K): δ = 8.31 (d, 1H, H5, *J* = 9.0), 7.95–8.01 (m, 2H, H2', H6'), 7.73 (d, 1H, H8, *J* = 2.8), 7.49 (t, 2H, H3', H5', *J* = 7.7), 7.38 (t, 1H, H4', *J* = 7.4), 7.17 (dd, 1H, H6, *J* = 9.0, *J* = 2.6), 3.85 (s, 3H, OCH_3_). Anal. Calcd. for C_16_H_11_BrN_2_OS: C, 53.49; H, 3.09; N, 7.8. Found: C, 53.45; H, 3.05; N, 7.75.

### Refinement

All hydrogen atoms were placed in the calculated positions, with C—H = 0.95 Å (CH-groups) and 0.98 Å (CH_3_-group), and refined in the riding model, with *U*_iso_(H) = 1.2–1.5*U*_eq_(C).

### Crystal data

**Table:**

C_16_H_11_BrN_2_OS	*F*(000) = 720
*M**_r_* = 359.24	*D*_x_ = 1.763 Mg m^−^^3^
Monoclinic, *P*2_1_/*n*	Mo *K*α radiation, λ = 0.71073 Å
Hall symbol: -P 2yn	Cell parameters from 9814 reflections
*a* = 3.8346 (4) Å	θ = 2.2–30.5°
*b* = 9.4848 (11) Å	µ = 3.19 mm^−^^1^
*c* = 37.236 (4) Å	*T* = 100 K
β = 91.810 (2)°	Needle, colourless
*V* = 1353.6 (3) Å^3^	0.35 × 0.15 × 0.15 mm
*Z* = 4	

### Data collection

**Table:**

Bruker APEXII CCD diffractometer	3924 independent reflections
Radiation source: fine-focus sealed tube	3578 reflections with *I* > 2σ(*I*)
Graphite monochromator	*R*_int_ = 0.041
φ and ω scans	θ_max_ = 30.0°, θ_min_ = 2.2°
Absorption correction: multi-scan (*SADABS*; Sheldrick, 2003)	*h* = −5→5
*T*_min_ = 0.402, *T*_max_ = 0.646	*k* = −13→13
17224 measured reflections	*l* = −52→52

### Refinement

**Table:**

Refinement on *F*^2^	Primary atom site location: structure-invariant direct methods
Least-squares matrix: full	Secondary atom site location: difference Fourier map
*R*[*F*^2^ > 2σ(*F*^2^)] = 0.032	Hydrogen site location: inferred from neighbouring sites
*wR*(*F*^2^) = 0.070	H-atom parameters constrained
*S* = 1.00	*w* = 1/[σ^2^(*F*_o_^2^) + (0.0196*P*)^2^ + 2.89*P*] where *P* = (*F*_o_^2^ + 2*F*_c_^2^)/3
3924 reflections	(Δ/σ)_max_ = 0.001
191 parameters	Δρ_max_ = 0.59 e Å^−^^3^
0 restraints	Δρ_min_ = −0.95 e Å^−^^3^

### Special details

**Table:**

Geometry. All e.s.d.'s (except the e.s.d. in the dihedral angle between two l.s. planes) are estimated using the full covariance matrix. The cell e.s.d.'s are taken into account individually in the estimation of e.s.d.'s in distances, angles and torsion angles; correlations between e.s.d.'s in cell parameters are only used when they are defined by crystal symmetry. An approximate (isotropic) treatment of cell e.s.d.'s is used for estimating e.s.d.'s involving l.s. planes.
Refinement. Refinement of *F*^2^ against ALL reflections. The weighted *R*-factor *wR* and goodness of fit *S* are based on *F*^2^, conventional *R*-factors *R* are based on *F*, with *F* set to zero for negative *F*^2^. The threshold expression of *F*^2^ > σ(*F*^2^) is used only for calculating *R*-factors(gt) *etc*. and is not relevant to the choice of reflections for refinement. *R*-factors based on *F*^2^ are statistically about twice as large as those based on *F*, and *R*- factors based on ALL data will be even larger.

### Fractional atomic coordinates and isotropic or equivalent isotropic displacement parameters (Å^2^)

**Table:**

	*x*	*y*	*z*	*U*_iso_*/*U*_eq_	
Br1	−0.02456 (5)	0.18794 (2)	0.820287 (5)	0.01435 (6)	
O1	0.7765 (4)	−0.18472 (16)	0.97119 (4)	0.0169 (3)	
N1	0.3136 (5)	0.49917 (19)	0.88973 (5)	0.0132 (3)	
C2	0.1687 (5)	0.4553 (2)	0.85658 (5)	0.0128 (4)	
C3	0.1626 (5)	0.3103 (2)	0.85500 (5)	0.0132 (4)	
N4	0.3079 (4)	0.26156 (18)	0.88734 (4)	0.0114 (3)	
C4A	0.4010 (5)	0.1337 (2)	0.90429 (5)	0.0108 (3)	
C5	0.3563 (5)	−0.0040 (2)	0.89273 (5)	0.0122 (4)	
H5	0.2447	−0.0235	0.8701	0.015*	
C6	0.4774 (5)	−0.1141 (2)	0.91473 (6)	0.0140 (4)	
H6	0.4476	−0.2091	0.9071	0.017*	
C7	0.6429 (5)	−0.0847 (2)	0.94802 (5)	0.0123 (4)	
C8	0.6842 (5)	0.0541 (2)	0.96019 (5)	0.0131 (4)	
H8	0.7929	0.0739	0.9829	0.016*	
C8A	0.5618 (5)	0.1615 (2)	0.93805 (5)	0.0122 (4)	
S9	0.59204 (13)	0.34210 (5)	0.947997 (13)	0.01341 (10)	
C9A	0.3926 (5)	0.3811 (2)	0.90637 (5)	0.0120 (4)	
C10	0.0582 (5)	0.5587 (2)	0.82933 (5)	0.0130 (4)	
C11	−0.0888 (5)	0.6869 (2)	0.83961 (6)	0.0154 (4)	
H11	−0.1203	0.7057	0.8644	0.018*	
C12	−0.1889 (6)	0.7866 (2)	0.81419 (6)	0.0192 (4)	
H12	−0.2904	0.8729	0.8216	0.023*	
C13	−0.1415 (6)	0.7611 (2)	0.77781 (6)	0.0198 (4)	
H13	−0.2092	0.8298	0.7604	0.024*	
C14	0.0052 (6)	0.6347 (3)	0.76713 (6)	0.0189 (4)	
H14	0.0374	0.6168	0.7423	0.023*	
C15	0.1053 (5)	0.5339 (2)	0.79261 (6)	0.0159 (4)	
H15	0.2062	0.4477	0.7851	0.019*	
C16	0.7532 (6)	−0.3284 (2)	0.96000 (6)	0.0161 (4)	
H16A	0.8656	−0.3888	0.9783	0.024*	
H16B	0.8708	−0.3400	0.9372	0.024*	
H16C	0.5073	−0.3551	0.9568	0.024*	

### Atomic displacement parameters (Å^2^)

**Table:**

	*U*^11^	*U*^22^	*U*^33^	*U*^12^	*U*^13^	*U*^23^
Br1	0.01465 (9)	0.01602 (10)	0.01221 (9)	−0.00152 (7)	−0.00252 (6)	−0.00246 (7)
O1	0.0232 (7)	0.0110 (7)	0.0161 (7)	0.0009 (6)	−0.0046 (6)	0.0000 (5)
N1	0.0148 (8)	0.0134 (8)	0.0114 (7)	−0.0011 (6)	0.0001 (6)	−0.0007 (6)
C2	0.0129 (8)	0.0144 (9)	0.0111 (8)	−0.0006 (7)	0.0002 (7)	−0.0001 (7)
C3	0.0132 (8)	0.0143 (9)	0.0121 (8)	−0.0011 (7)	−0.0012 (7)	−0.0025 (7)
N4	0.0121 (7)	0.0119 (8)	0.0102 (7)	0.0002 (6)	−0.0009 (6)	−0.0007 (6)
C4A	0.0097 (8)	0.0134 (9)	0.0091 (8)	−0.0004 (7)	−0.0005 (6)	0.0004 (7)
C5	0.0109 (8)	0.0141 (9)	0.0116 (8)	−0.0005 (7)	−0.0015 (7)	−0.0014 (7)
C6	0.0130 (9)	0.0117 (9)	0.0171 (9)	−0.0011 (7)	−0.0006 (7)	−0.0016 (7)
C7	0.0120 (8)	0.0138 (9)	0.0111 (8)	−0.0003 (7)	0.0004 (7)	0.0018 (7)
C8	0.0136 (8)	0.0149 (9)	0.0107 (8)	−0.0020 (7)	0.0001 (7)	−0.0007 (7)
C8A	0.0114 (8)	0.0125 (9)	0.0127 (8)	−0.0008 (7)	−0.0001 (7)	−0.0015 (7)
S9	0.0176 (2)	0.0104 (2)	0.0120 (2)	−0.00075 (17)	−0.00341 (17)	−0.00098 (16)
C9A	0.0124 (8)	0.0129 (9)	0.0106 (8)	−0.0018 (7)	−0.0006 (7)	−0.0020 (7)
C10	0.0109 (8)	0.0157 (9)	0.0125 (8)	−0.0019 (7)	−0.0016 (7)	0.0012 (7)
C11	0.0148 (9)	0.0150 (9)	0.0163 (9)	−0.0012 (8)	0.0001 (7)	0.0002 (7)
C12	0.0168 (10)	0.0143 (10)	0.0262 (11)	0.0000 (8)	−0.0028 (8)	0.0027 (8)
C13	0.0168 (10)	0.0213 (10)	0.0210 (10)	−0.0025 (8)	−0.0032 (8)	0.0092 (8)
C14	0.0186 (10)	0.0250 (11)	0.0130 (9)	−0.0025 (9)	−0.0020 (8)	0.0044 (8)
C15	0.0130 (8)	0.0184 (10)	0.0164 (9)	−0.0005 (7)	0.0005 (7)	0.0031 (8)
C16	0.0188 (9)	0.0112 (9)	0.0182 (9)	0.0008 (8)	−0.0022 (8)	−0.0009 (7)

### Geometric parameters (Å, º)

**Table:**

Br1—C3	1.864 (2)	C8—H8	0.9500
O1—C7	1.371 (2)	C8A—S9	1.756 (2)
O1—C16	1.427 (2)	S9—C9A	1.746 (2)
N1—C9A	1.311 (3)	C10—C11	1.398 (3)
N1—C2	1.401 (2)	C10—C15	1.404 (3)
C2—C3	1.377 (3)	C11—C12	1.384 (3)
C2—C10	1.465 (3)	C11—H11	0.9500
C3—N4	1.390 (2)	C12—C13	1.394 (3)
N4—C9A	1.370 (3)	C12—H12	0.9500
N4—C4A	1.408 (3)	C13—C14	1.388 (3)
C4A—C5	1.385 (3)	C13—H13	0.9500
C4A—C8A	1.407 (3)	C14—C15	1.392 (3)
C5—C6	1.397 (3)	C14—H14	0.9500
C5—H5	0.9500	C15—H15	0.9500
C6—C7	1.403 (3)	C16—H16A	0.9800
C6—H6	0.9500	C16—H16B	0.9800
C7—C8	1.399 (3)	C16—H16C	0.9800
C8—C8A	1.383 (3)		
			
C7—O1—C16	117.30 (16)	C9A—S9—C8A	89.72 (10)
C9A—N1—C2	104.00 (17)	N1—C9A—N4	114.52 (17)
C3—C2—N1	109.89 (17)	N1—C9A—S9	133.52 (16)
C3—C2—C10	129.46 (18)	N4—C9A—S9	111.95 (15)
N1—C2—C10	120.63 (18)	C11—C10—C15	118.53 (19)
C2—C3—N4	106.81 (17)	C11—C10—C2	120.19 (18)
C2—C3—Br1	131.09 (16)	C15—C10—C2	121.25 (19)
N4—C3—Br1	121.94 (15)	C12—C11—C10	120.8 (2)
C9A—N4—C3	104.77 (17)	C12—C11—H11	119.6
C9A—N4—C4A	115.35 (16)	C10—C11—H11	119.6
C3—N4—C4A	139.80 (17)	C11—C12—C13	120.3 (2)
C5—C4A—N4	130.27 (17)	C11—C12—H12	119.9
C5—C4A—C8A	120.03 (18)	C13—C12—H12	119.9
N4—C4A—C8A	109.70 (17)	C14—C13—C12	119.7 (2)
C4A—C5—C6	119.16 (18)	C14—C13—H13	120.2
C4A—C5—H5	120.4	C12—C13—H13	120.2
C6—C5—H5	120.4	C13—C14—C15	120.2 (2)
C5—C6—C7	120.13 (19)	C13—C14—H14	119.9
C5—C6—H6	119.9	C15—C14—H14	119.9
C7—C6—H6	119.9	C14—C15—C10	120.5 (2)
O1—C7—C8	114.26 (17)	C14—C15—H15	119.8
O1—C7—C6	124.62 (18)	C10—C15—H15	119.8
C8—C7—C6	121.12 (18)	O1—C16—H16A	109.5
C8A—C8—C7	117.81 (18)	O1—C16—H16B	109.5
C8A—C8—H8	121.1	H16A—C16—H16B	109.5
C7—C8—H8	121.1	O1—C16—H16C	109.5
C8—C8A—C4A	121.72 (18)	H16A—C16—H16C	109.5
C8—C8A—S9	125.00 (16)	H16B—C16—H16C	109.5
C4A—C8A—S9	113.27 (15)		
			
C9A—N1—C2—C3	0.9 (2)	N4—C4A—C8A—C8	−178.98 (18)
C9A—N1—C2—C10	−177.60 (18)	C5—C4A—C8A—S9	−179.60 (15)
N1—C2—C3—N4	−0.5 (2)	N4—C4A—C8A—S9	0.2 (2)
C10—C2—C3—N4	177.8 (2)	C8—C8A—S9—C9A	178.51 (19)
N1—C2—C3—Br1	174.83 (15)	C4A—C8A—S9—C9A	−0.68 (16)
C10—C2—C3—Br1	−6.9 (4)	C2—N1—C9A—N4	−0.9 (2)
C2—C3—N4—C9A	0.0 (2)	C2—N1—C9A—S9	177.88 (18)
Br1—C3—N4—C9A	−175.90 (14)	C3—N4—C9A—N1	0.6 (2)
C2—C3—N4—C4A	−176.4 (2)	C4A—N4—C9A—N1	178.04 (18)
Br1—C3—N4—C4A	7.7 (3)	C3—N4—C9A—S9	−178.45 (14)
C9A—N4—C4A—C5	−179.7 (2)	C4A—N4—C9A—S9	−1.0 (2)
C3—N4—C4A—C5	−3.5 (4)	C8A—S9—C9A—N1	−177.9 (2)
C9A—N4—C4A—C8A	0.5 (2)	C8A—S9—C9A—N4	0.95 (15)
C3—N4—C4A—C8A	176.6 (2)	C3—C2—C10—C11	146.1 (2)
N4—C4A—C5—C6	179.2 (2)	N1—C2—C10—C11	−35.7 (3)
C8A—C4A—C5—C6	−1.0 (3)	C3—C2—C10—C15	−35.6 (3)
C4A—C5—C6—C7	−0.2 (3)	N1—C2—C10—C15	142.5 (2)
C16—O1—C7—C8	−178.10 (18)	C15—C10—C11—C12	0.6 (3)
C16—O1—C7—C6	1.8 (3)	C2—C10—C11—C12	178.9 (2)
C5—C6—C7—O1	−178.68 (19)	C10—C11—C12—C13	−0.6 (3)
C5—C6—C7—C8	1.2 (3)	C11—C12—C13—C14	0.3 (3)
O1—C7—C8—C8A	178.87 (18)	C12—C13—C14—C15	−0.1 (3)
C6—C7—C8—C8A	−1.1 (3)	C13—C14—C15—C10	0.2 (3)
C7—C8—C8A—C4A	−0.1 (3)	C11—C10—C15—C14	−0.4 (3)
C7—C8—C8A—S9	−179.27 (15)	C2—C10—C15—C14	−178.7 (2)
C5—C4A—C8A—C8	1.2 (3)		

### Hydrogen-bond geometry (Å, º)

**Table:**

*D*—H···*A*	*D*—H	H···*A*	*D*···*A*	*D*—H···*A*
C8—H8···O1^i^	0.95	2.56	3.465 (3)	158

Symmetry code: (i) −*x*+2, −*y*, −*z*+2.
